# A new type of two-dimensional carbon crystal prepared from 1,3,5-trihydroxybenzene

**DOI:** 10.1038/srep40796

**Published:** 2017-01-17

**Authors:** Qi-Shi Du, Pei-Duo Tang, Hua-Lin Huang, Fang-Li Du, Kai Huang, Neng-Zhong Xie, Si-Yu Long, Yan-Ming Li, Jie-Shan Qiu, Ri-Bo Huang

**Affiliations:** 1 State key Laboratory of Bioenergy Enzyme Technology, National Engineering Research Center for Non-food Biorefinery, Guangxi Academy of Sciences, Nanning, Guangxi 530007, China; 2 Gordon Life Science Institute, 53 South Cottage Road, Belmont, MA, 02478, USA; 3 Institute of Carbon Materials, Dalian University of Technology, No.2 Linggong Road, Ganjingzi District, Dalian, Liaoning, 116024, China

## Abstract

A new two-dimensional (2D) carbon crystal, different from graphene, has been prepared from 1,3,5-trihydroxybenzene, consisting of 4-carbon and 6-carbon rings in 1:1 ratio, named 4–6 carbophene by authors, in which all carbon atoms possess sp^2^ hybrid orbitals with some distortion, forming an extensive conjugated π-bonding planar structure. The angles between the three σ-bonds of the carbon sp^2^ orbitals are roughly 120°, 90°, and 150°. Each of the three non-adjacent sides of a 6C-ring is shared with a 4C-ring; and each of the two opposite sides of a 4C-ring is shared with a 6C-ring. Dodecagonal holes with a diameter of approximate 5.8 Å are regularly located throughout the 2D carbon crystal. Even though the bond energies in 4–6 carbophene are weaker than those in the graphene, the new planar crystal is quite stable in ambient conditions. The 4–6 carbophene can be synthetized from 1,3,5-trihydroxybenzene or other benzene derivatives through dehydration and polymerization reactions, and may possess several possible patterns that form a family of 2D carbon crystals. A possible side reaction involving 1,3,5-trihydroxybenzene is also discussed, which may produce a carbon-oxygen two dimensional crystal.

Graphene^1^,^2^ is an allotrope of elemental carbon in the form of an atomic-scale, two-dimensional, honey-comb lattice, in which all carbon atoms are in sp^2^ electron configuration, resulting in an extensive conjugated π-system. Graphene is the basic structural element of several other carbon allotropes, including graphite^3^,^4^, carbon nanotubes^5^ and fullerenes (C_60_)^6^. Graphene is an excellent material possessing many outstanding physical and chemical properties, such as high electrical and thermal conductivity^7^, huge specific surface area^8^, good optical transparency^2^, and excellent mechanical strength^9^,^10^. Therefore, graphene is expected to have many applications in broad areas^11^,^12^.

From material science and structural chemistry viewpoints, graphene is a two-dimensional (2D) crystal and a 2D carbon material^13^. Since the first 2D carbon crystal graphene was found in 2004^1^,^2^, several other 2D crystals and materials of carbon have emerged^14^,^15^. In recent years the 2D crystals and materials become a booming research field. A comprehensive review on this field can be found in a review article by Peng *et al*.^16^. Chemically, a sheet of graphene is a large aromatic molecule consisting of hexagonal planar structural units^17^,^18^ that are similar to anthracene and phenanthrene. In graphene, all carbon atoms are in standard sp^2^ electronic configuration so that the angles between the three σ-bonds of carbon sp^2^ orbitals are 120°, 120°, and 120°, just as they are in the carbon atoms in benzene and other aromatic molecules^19^,^20^.

Actually, the orbitals in carbon atoms can also be in a distorted sp^2^ hybrid form, resulting in planar structural units other than hexagons^21^,^22^,^23^,^24^. This possibility raises an interesting question: “Are there other two-dimensional carbon crystals that are different from graphene?” We have explored this question from quantum chemical theory and synthetic chemistry.

In this paper, we report on a new 2D carbon crystal that has been prepared from 1,3,5-trihydroxybenzene through dehydration and polymerization reactions. The new type of 2D carbon crystals may exist in several possible forms, resulting in a 2D family of closely related crystals.

## Theory and Method

**Figure 1:**
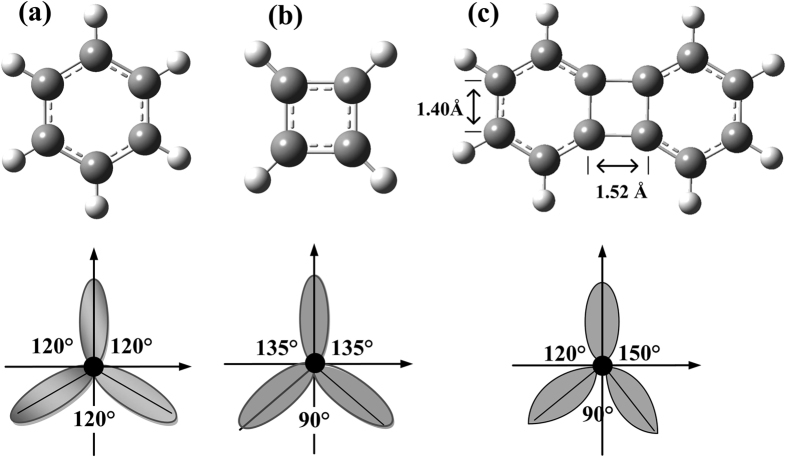
The chemical structures and carbon sp^2^ hybrid orbitals in conjugate π-molecules. (**a**) In benzene the carbon atoms are in standard sp^2^ hybrid orbital, in which the three angles between the three σ-bonds are 120°, 120°, and 120°. (**b**) In cyclobutadiene the carbon atoms are in distorted sp^2^ hybrid orbital, in which the three angles between the three σ-bonds are 90°, 135°, and 135°. (**c**) Biphenylene consists of two benzene rings joined together by a cyclobutadiene, thus forming a 6-4-6 arene system. In biphenylene the four carbon atoms in the square ring are in distorted sp^2^ hybrid orbital, in which the three angles between the three σ-bonds are 90°, 120°, and 150°.

The carbon atoms of benzene have standard sp^2^ hybrid orbitals, in which the three σ-bonds form three equal angles 120°, 120°, and 120°, as shown in Fig. 1(a). In contrast, in cyclobutadiene, the carbon atoms have distorted sp^2^ hybrid orbitals, so that the three angles between the three σ-bonds are 90°, 135°, and 135°, shown in Fig. 1(b). Biphenylene is a well-studied planar molecule, quite stable both experimentally and thermodynamically^25^,^26^, which is a polycyclic hydrocarbon, composed of two benzene rings joined together by a cyclobutadiene, thus forming a 6-4-6 arene system. Each of the four carbon atoms in the square ring cyclobutadiene moiety of biphenylene has distorted sp^2^ hybrid orbitals and associated σ-bond angles of 120°, 90°, and 150°, as shown in Fig. 1(c). A number of higher polycyclics containing the biphenylene nuclei were prepared and studied in 1970 s and 1980s^27^,^28^,^29^,^30^,^31^,^32^. These more complex molecules, containing several biphenylene structures, are actually small pieces of 2D carbon crystals. Therefore, we believed that it should be possible to construct new 2D carbon crystals, different from graphene, using the carbon atoms with these distorted sp^2^ electron orbitals.

**Figure 2:**
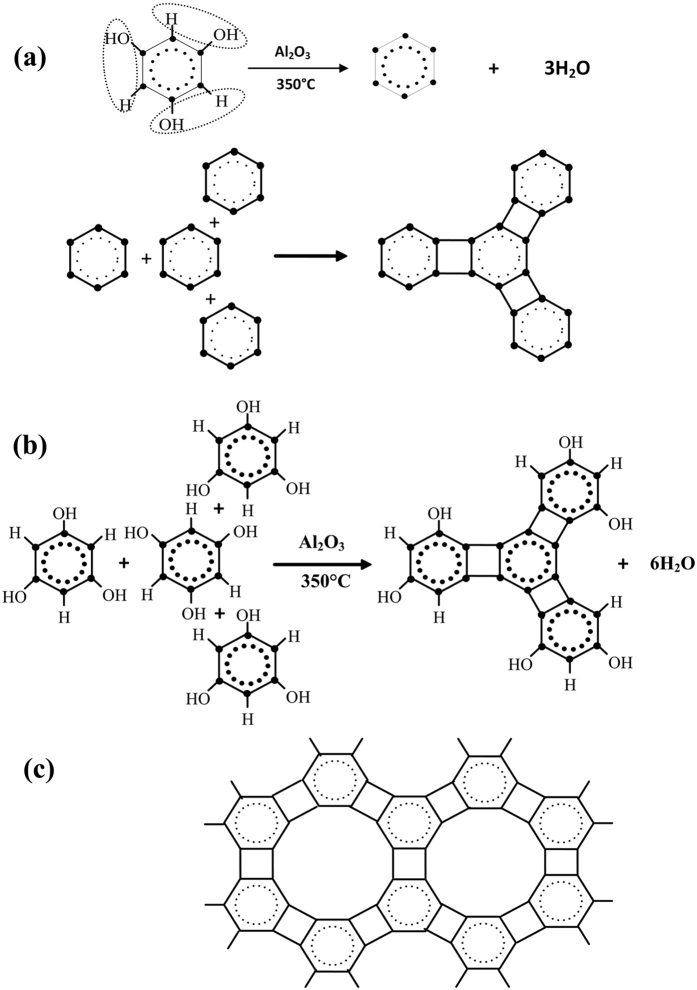
Illustration of chemical reaction mechanism from 1,3,5-trihydroxybenzene to 4–6 carbophene 2D crystal. (**a**) The polymerization reaction could happen through intra-molecular dehydration of 1,3,5-trihydroxybenzene. (**b**) The polymerization reaction also could happen through inter-molecular dehydration between 1,3,5-trihydroxybenzene molecules. (**c**) With the joining of more 1,3,5-trihydroxybenzene molecules the fragments of 2D carbon crystal grows up quickly.

The overall chemical reactions in the synthesis of new 2D carbon crystal are outlined in Fig. 2. The polymerization reaction could happen through intra-molecular dehydration of 1,3,5-trihydroxybenzene, shown in Fig. 2(a). After three water molecules are stripped from a 1,3,5-trihydroxybenzene molecule by dehydrant aluminum oxide (γ-Al_2_O_3_), the bare 6 C rings (benzynes) combine with each other, forming a small fragment of the 2D carbon crystal. The polymerization reaction also could occur through inter-molecular dehydration between 1,3,5-trihydroxybenzene molecules, as shown in Fig. 2(b). With the joining of more 1,3,5-trihydroxybenzene molecules the fragments of 2D carbon crystal grow quickly (Fig. 2c).

Figure 2 is only a simple illustration of the overall chemical reactions of 1,3,5-trihydroxybenzene that can lead to a 4–6 carbophene 2D crystal. The actual reaction mechanism may be much more complex. The dehydration reaction of 1,3,5-trihydroxybenzene may occur in several steps, losing one, two, and three water molecules step-by-step, forming different intermediate arynes (or benzynes) in the process^33^,^34^. In addition, the dehydration and polymerization reactions of 1,3,5-trihydroxybenzene could be accompanied by different side reactions, yielding by-products under certain conditions. One of such side reaction is studied in next section, which produces a benzene-ether 2D crystal, a by-product.

In this study, the synthesis of the new crystals were performed in a quartz glass tube furnace in an argon atmosphere at a temperatures of 350–380 °C using γ-aluminum oxide (γ-Al_2_O_3_) as the dehydrant. Detailed experimental operations and conditions are included in the Supplementary Information (SI-1).

## Results

### QM calculations of reaction Gibbs free energies

The inner energies (Δ*U°*_R_), enthalpies (Δ*H°*_R_), and Gibbs free energies (Δ*G°*_R_) of the synthesis reactions for the new 2D carbon crystal have been calculated using DFT method B3LYP/6-311 + G(d,p)^31^,^32^,^33^,^34^ for two possible reaction pathways. In the first pathway, two water molecules are removed from two 1,3,5-trihydroxybenzene molecules, producing a biphenylene molecule.


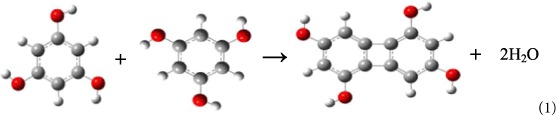


Based on QM calculations, the Gibbs free energy of this reaction at 350 °C (T = 623 K) is positive; i.e., Δ*G°*_R1_1_ = 28.84 kCal/mol. In the next step, the water molecules are absorbed by the dehydrant, γ-aluminum oxide:


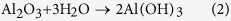


The Gibbs free energy for the adsorption of one mole water molecule is calculated to be negative; i.e., Δ*G°*_R1_2_ = −66.02 kCal/mol. Therefore, the total Gibbs free energy of the entire first reaction pathway is Δ*G°*_R1_ = Δ*G°*_R1_1_ + Δ*G°*_R1_2_ = 28.84 + −66.02 × 2 = −103.19 kCal/mol; i.e., significantly negative and thermodynamically spontaneous.

In the second reaction pathway, one water molecule is removed from two 1,3,5-trihydroxybenzene molecules, producing a benzene-ether molecule:


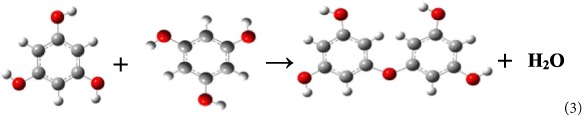


In this first step of the reaction, the Gibbs free energy is positive, Δ*G°*_R2_1_ = 7.49 kCal/mol. In the second step, when one mole of water is absorbed by aluminum oxide (Al_2_O_3_), the reaction Gibbs free energy Δ*G°*_R2_2_ = −66.02 kCal/mol. As a result, the total Gibbs free energy of the second pathway is Δ*G°*_R2_ = Δ*G°*_R2_1_ + Δ*G°*_R2_2_ = 7.49–66.02 = −58.53 kCal/mol.

Therefore, the total Gibbs free energy is more favourable for the first reaction pathway (Δ*G°*_R1_ = −103.19 kCal/mol) than for the second pathway (Δ*G°*_R2_ = −58.53 kCal/mol). However, the former pathway requires a higher reaction temperature than the latter, because of the higher reaction enthalpy in the first step (ΔH*°*_R1_1_ = 46.52 kCal/mol, compared to ΔH*°*_R2_1_ = 11.40 kCal/mol).

The QM calculation results are summarized in Table 1, and the detailed QM calculation results can be found in Supplementary Information (SI-2 and SI-3).

### Crystal structure of the 4–6 carbophene

**Figure 3:**
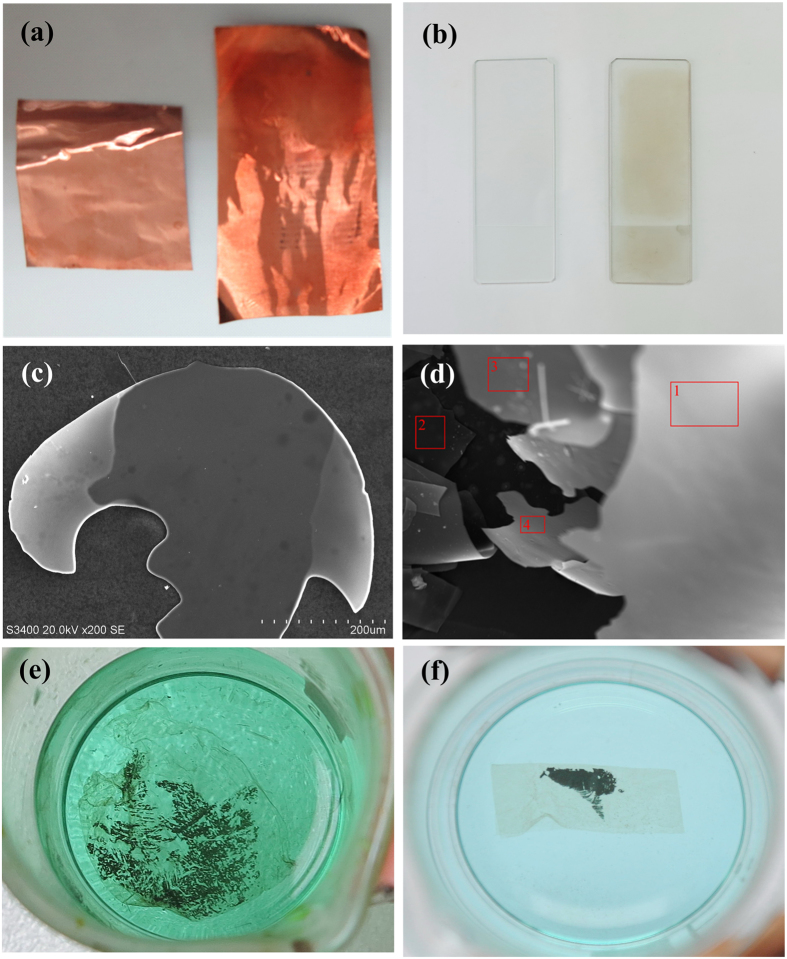
The membranes of 2D carbon crystal, coated on the quartz glass sheet and copper foil. (**a**) Comparison of copper foil before (left) and after (right) the 4–6 carbophene film was coated. (**b**) Comparison of quartz glass sheet before (left) and after (right) the 4–6 carbophene film was coated. (**c**) The SEM (scanning electron microscope) image of the 2D carbon crystal film separated from copper foil. (**d**) The SEM image of the 2D carbon crystal film separated from quartz glass sheet. After the carbon films of the 2D crystal are separated from the copper foil and quartz glass sheet, transparent carbon films of 4–6 carbophene are obtained. (**e**,**f**) The 4–6 carbophene films separated from copper foil.

The membranes of 2D carbon crystal, formed on copper foil and on a quartz glass sheet, are shown in Fig. 3(a) and (b). When the 4–6 films are removed from the glass sheet using N,N-dimethyl formamide, transparent carbon films are obtained. The SEM (scanning electron microscopy) images^35^ of the films are shown in Fig. 3(c) and (d). After the copper foils are dissolved in FeCl_3_ + HCl solution completely, the films of the 4–6 carbophene were separated, as shown in Fig. 3(e) and (f).

The numbered red squares on the 4–6 carbophene film separated from the quatz glass sheet (Fig. 3(d)) indicate the locations where the element analyses were performed using the EDS (energy dispersive spectrometry)^36^ provided by the SEM instrument (IXRF, 550i). The element components in the 4–6 carbophene are listed in Table 2, showing that carbon is the dominant element in the films. Other elements (oxygen and silicon) exist only in trace quantities, which may originate from the quartz glass. These data confirm that the 2D carbon crystal is a carbon allotrope.

**Figure 4:**
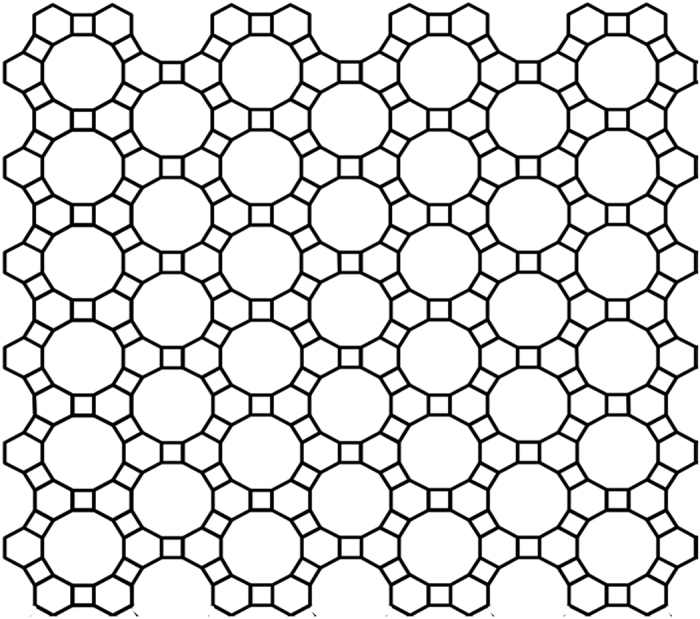
The structural patter of the new 2D carbon crystal prepared from 1,3,5-trihydroxybenzene. The 2D carbon crystal consists of 4-carbon rings and 6-carbon rings in ratio 1:1. The side lengths of the six-carbon rings are around 1.42~1.46 Å, and the two bonds, joining the two six-carbon rings, are around 1.50~1.52 Å. In the 2D carbon crystal dodecagon holes with diameter around 5.8 Å are regularly located. The 2D carbon crystal has two types of edge structural patterns. In one edge pattern the grooves are separated by six-ring spur; and in the other pattern the grooves are separated by spur of (6C-ring)-(4C-ring)-(6C-ring).

The 2D carbon crystal consists of four-carbon rings and six-carbon rings, in which the three non-bordering sides of a six-carbon ring are shared by three four-carbon rings; and the two opposite sides of a four-carbon ring are shared by two six-carbon rings. The structure of the 2D carbon crystal is shown in Fig. 4. The side lengths of the six-carbon rings are around 1.42~1.46 Å, and the two bonds, joining the two six-carbon rings, are around 1.50~1.52 Å. In the 2D carbon crystal dodecagon holes with diameter around 5.8 Å are regularly located. The 2D crystal of 4–6 carbophene has two types of edge structural patterns. In one edge pattern the grooves are separated by 6C-ring spur; and in the other pattern the grooves are separated by spur of (6C-ring)-(4C-ring)-(6C-ring).

### XRD pattern of 4–6 carbophene crystal

**Figure 5:**
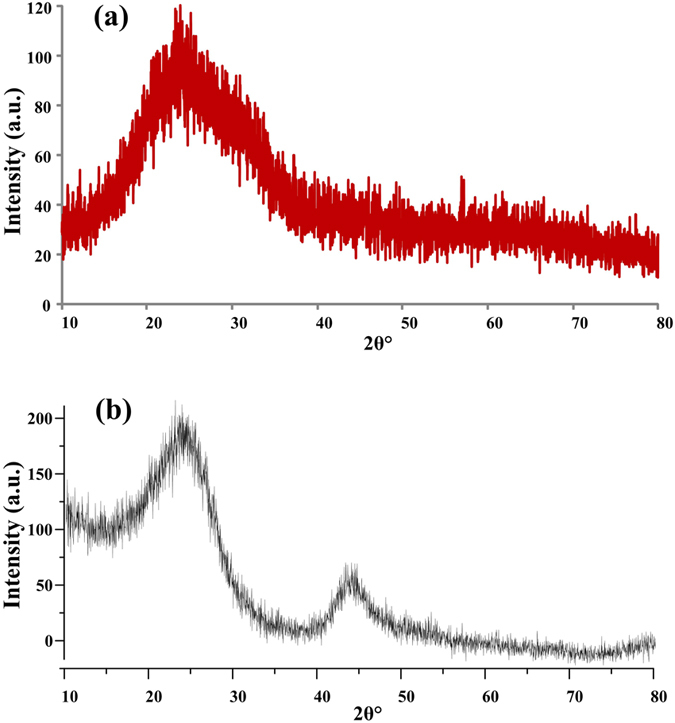
The X-ray diffraction (XRD) pattern of the new 2D carbon crystal. (**a**) The XRD pattern of 4–6 carbophene has only one peak, indicating a few-layer structure. The position of diffraction angle in the 4–6 carbophene 2D carbon crystal is around 2θ = ~23°. (**b**) The XRD pattern of reduced graphene oxide (rGO). The position of diffraction angle of rGO is at 2θ = ~26°. In graphene the interlayer distance (d_002_) is 0.343 nm for the AB-stack. The estimated interlayer distance in the new 2D carbon crystal is 0.387 nm. The smaller 2θ and larger d_002_ may be caused by the distorted sp^2^ hybrid orbitals of carbon atoms in the 4–6 carbophene crystal.

The X-ray diffraction (XRD) pattern of the new carbon crystal (Fig. 5a) has only one peak that is not very sharp, similar to the XRD pattern of reduced graphene oxide (rGO)^37^,^38^, shown in Fig. 5(b), indicating a few-layer structure. However, the position of diffraction angle (2θ) in the 4–6 carbophene 2D crystal is around 2θ = ~23°, not the 2θ = ~26° observed in the XRD of graphene^39^. In graphene, the interlayer distance (d_002_) is 0.343 nm for the AB-stack^40^. Based on the XRD equation


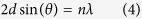


estimated interlayer distance in the 4–6 carbophene is 0.387 nm. The larger d_002_ and smaller 2θ may be caused by the weaker van der Waals interaction between the carbon layers that results from the distorted sp^2^ hybrid orbitals of carbon atoms in the 4–6 carbophene.

### Raman spectrum of 4–6 carbophene 2D crystal

**Figure 6:**
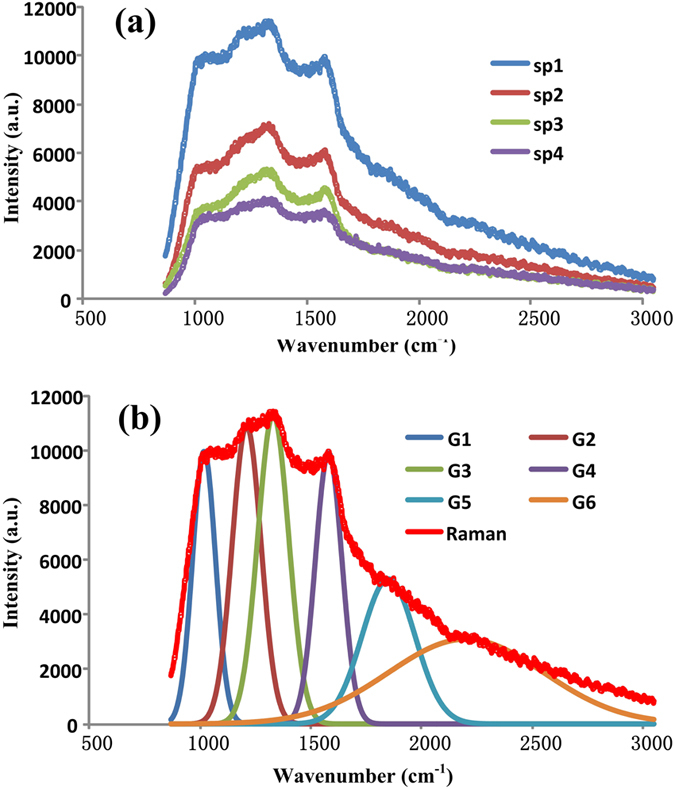
Raman spectrum of the new 2D carbon crystal. (**a**) The new 2D carbon crystal has much more complicated symmetrical structure than that of graphere. Graphene only possesses hexagonal symmetry. In contrast in the 4–6 carbophene there are 4-carbon rings, 6-carbon rings, large dodecagon holes, and two types of edges. The shape of Raman spectrum of the 4–6 carbophene is like a rock with a steep left side, a broad and cragged top, and a slope right side. (**b**) Raman peaks involved in the Raman spectrum of new 2D carbon crystal. Simulation and fitting calculations reveal that the Raman spectrum of the 4–6 carbonphene crystal consists of six gauss peaks.

The 2D carbon crystal has a much more complicated symmetrical structure than does graphene. Graphene only possesses the hexagonal symmetry. In contrast in the 4–6 carbophene there are 4-carbon rings, 6-carbon rings, large dodecagonal holes, and two types of edges. The Raman spectrum of 4–6 carbophene is shown in Fig. 6, which was obtained using a laser beam with a 785 nm wavelength. Whenever the power of the laser beam was stronger, the 4–6 carbophene film broke, indicating a weaker bond energy in the new material than in graphene.

The shape of Raman spectrum of the 4–6 carbophene is very different from that of graphene^41^,^42^,^43^. The Raman spectrum of the 4–6 carbophene has a steep left side, a broad and cragged top, and a sloping right side, as shown in Fig. 6(a). Simulation and fitting calculations reveal that the Raman spectrum of the 4–6 carbophene crystal consists of six Gaussian peaks, as shown in Fig. 6(b). The wavenumbers and intensities of the six peaks are listed in Table 3. The peaks in the Raman spectrum of 4–6 carbophene need to be identified by experts.

### XPS spectra and analysis

**Figure 7:**
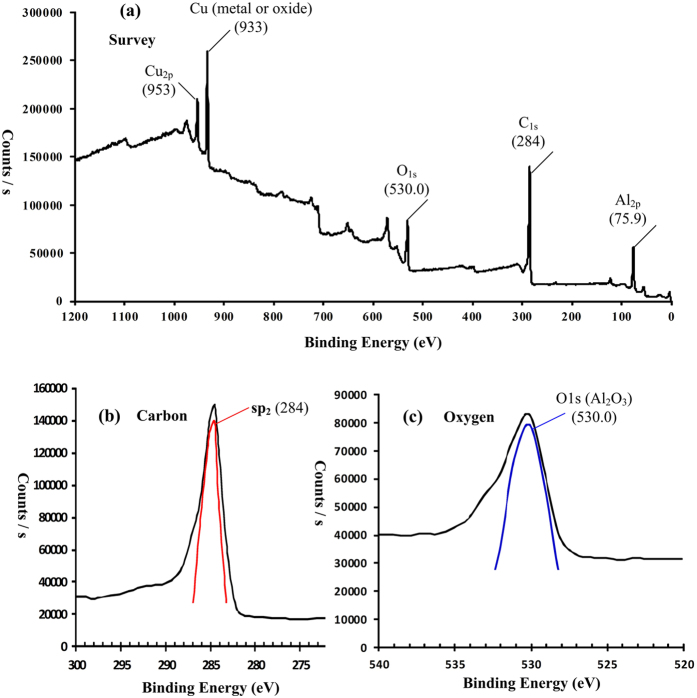
XPS (X-ray photoelectron spectroscopy) spectra of 4–6 carbophene. (**a**) Fully scanned survey of the 4–6 carbophene sample coated on copper foil. (**b**) High resolution XPS spectra of carbon (C1s) in 4–6 carbophene sample. The asymmetrical peak at 284.0 eV indicates that the carbon atoms are in sp^2^ electron configuration and compose π-π structure. (**c**) High resolution XPS spectra of oxygen (O1s) in 4–6 carbophene sample. The peak position of oxygen is at 530.0 eV, indicating the oxygen atoms are from oxides of aluminium and (or) copper, not from organic compounds.

The chemical composition of the 4–6 carbophene samples were further studied by XPS analysis^44^,^45^,^46^,^47^. Figure 7(a) is the fully XPS spectrum of the 2D crystal sample coated on copper foil, which shows peaks corresponding to elements C, Cu, Al, and O. The obvious source of the Cu peak is the copper foil, while the Al peak likely originated from the aluminum oxide dehydrant. However, the source of the oxygen is not clear. As mentioned above, two possible products could be produced from the reactant 1,3,5-trihydroxybenzene; one is 4–6 carbophene (containing C and H) and the other is the benzene-ether 2D crystal (containing, C, H, and O). The high resolution XPS spectrum of carbon is shown in Fig. 7(b), in which the asymmetrical peak at 284.0 eV indicates that the carbon atoms are in sp^2^ electron configuration and result in a π-π structure^46^. Figure 7(c) is the high resolution XPS spectrum of the oxygen signal. Usually the binding energies of oxygen atoms in metal oxides are in the range 529~530 eV, and the oxygen atoms in organic compounds are in the 531.5~532 eV range^42^. In Fig. 7(c) the peak position of oxygen is at 530.0 eV, indicating that the oxygen atoms are most likely from the oxides of aluminium and/or copper; i.e., not from organic compounds.

## Discussion

Based on quantum chemical considerations, the 2D planar crystals of carbon can only be composed of carbon atoms with sp^2^ electron configuration. In addition to the standard sp^2^ hybrid orbital of carbon atoms, which compose the hexagonal structure units, carbon atoms can also have distorted sp^2^ hybrid orbitals, such as those that have been reported in square^48^,^49^ or octagonal^50^,^51^ structural units, such as in cyclobutadiene, biphenylene^48^,^49^, and planar cyclooctatetraene^50^,^51^,^52^,^53^,^54^,^55^,^56^,^57^. In short, 2D carbon crystals, in addition to graphene, could be fabricated from carbon atoms with the distorted sp^2^ hybrid orbital.

During the synthesis, the 4–6 carbophene samples can be easily contaminated with the dehydrant aluminum oxide while vacuum pumping and purging with argon. Therefore, it was not possible to obtain AFM (atomic force microscopy) and STM (scanning tunneling microscope) images at atomic level. In order to obtain the high purity samples and high resolution AFM images that are needed, the synthesis methods and equipment will have to be improved.

However, despite the lack of such observations, we are confident that 4–6 carbophene was formed since the calculated Gibbs free energy of the reaction is quite negative (ΔG°_R1_ = −103.20 kCal/mol), indicating that its formation is thermodynamically favored. In addition, the XRD and XPS experiments provide indirect evidence of the structure and composition of the 4–6 carbophene.

**Figure 8:**
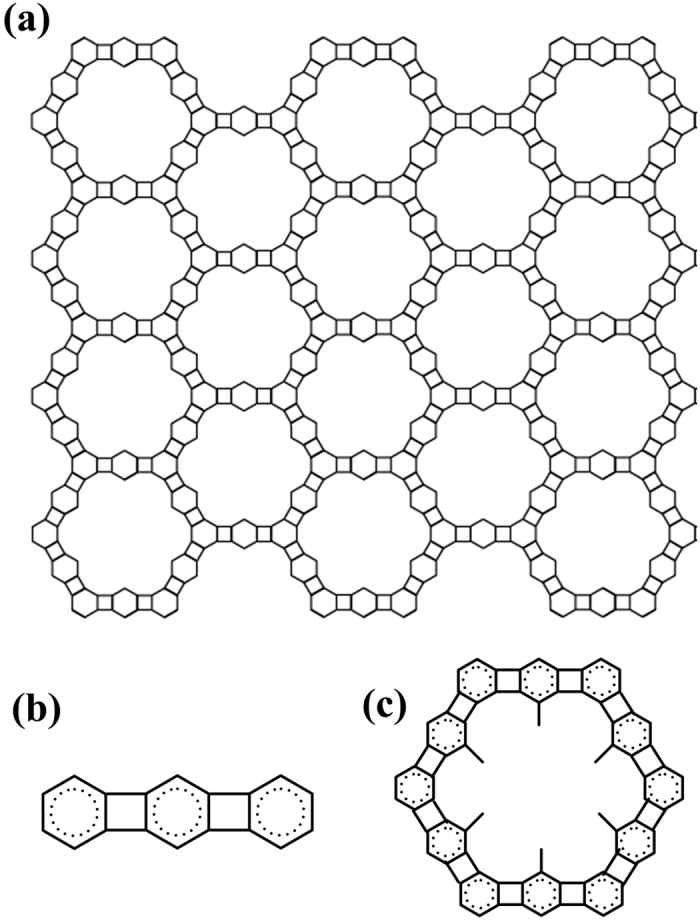
A possible 2D crystal pattern in the 4–6 carbophene family. (**a**) The 2D carbon crystal consists of joining hexagonal units. (**b**) Each side of the hexagonal unit is linearly ranged (6C-ring)-(4C-ring)-(6C-ring). (**c**) In each hexagonal hole there are six carbon vertexes, on which a single valence atom or atomic group is bonded.

The 2D crystal of 4–6 carbophene is less stable than graphene because of the distorted sp^2^ hybrid orbitals. However, it can exist in ambient conditions and may possess chemical and physical properties different from those of graphene. For example, the planer crystal may have a larger specific surface area and lower density than that of graphene because of the large dodecagonal holes in the 4–6 carbophene structure. Further, we expect that 4–6 carbophene may have several different 2D crystal patterns. For example, Fig. 8(a) shows a possibility of a larger hexagonal symmetrical structure resulting from combinations of short (6C-ring)-(4C-ring)-(6C-ring) segments (Fig. 8(b)). In addition, inside each hexagonal hole there are six vertex carbon atoms, to which a single valence atom or atomic group could be bonded (Fig. 8(c)).

In conclusion, the major findings from this study can be summarized as follows. (1) A new type of carbon 2D crystals has been constructed using planar 4-carbon rings and 6-carbon rings, which we have named “4–6 carbophenes”. (2) All carbon atoms in the 4–6 carbophenes are in sp^2^ electron configuration with some distortion, resulting in a large planar conjugated π-system. (3) The 4–6 carbophene may include several different 2D crystal patterns, forming a family of such crystals. (4) The 4–6 carbophenes can be synthetized through dehydration and polymerization reactions of 1,3,5-trihydroxybenzene or other benzene derivatives. (5) The 4–6 carbophenes are less stable than graphene because of the distorted sp^2^ orbitals; however, they can exist in ambient conditions and may possess chemical and physical properties that are different from those of graphene.

## Additional Information

**How to cite this article**: Du, Q.-S. *et al*. A new type of two-dimensional carbon crystal prepared from 1,3,5-trihydroxybenzene. *Sci. Rep.* **7**, 40796; doi: 10.1038/srep40796 (2017).

**Publisher's note:** Springer Nature remains neutral with regard to jurisdictional claims in published maps and institutional affiliations.

## Supplementary Material

Supplementary Information

Supplementary Information

## Figures and Tables

**Table 1:** The inner energies, enthalpy, and free energies of two possible reaction paths from 1,3,5-trihydroxybenzene (B3LYP/6-311 + G(d,p), 350 °C).

Molecules	ΔU° (a.u.)^a^	ΔH° (a.u.)	ΔG° (a.u.)
C_6_H_3_(OH)_3_	−457.751473	−457.749500	−457.866678
Product_1^b^	−762.648472	−762.646499	−762.811336
Product_2^c^	−839.102622	−839.100649	−839.283390
Al_2_O_3_	−710.521939	−710.519966	−710.609449
Al(OH)_3_	−470.026849	−470.024876	−470.119574
H_2_O	−76.389182	−76.387209	−76.438032
Reactions	ΔU° (kCal/mol)	ΔH° (kCal/mol)	ΔG° (kCal/mol)
Reaction__1_	−104.61	−107.49	−103.19
Step__1_1_	47.76	46.52	28.84
Step__1_2_	−152.36	−154.02	−132.03
Reaction__2_	−69.19	−65.60	−58.53
Step__2_1_	6.99	11.40	7.49
Step__2_2_	−76.18	−77.01	−66.02

^a^1 a.u. = 627.509469 kCal/mol = 2626.754637 kJ/mol.

^b^Product_1: Biphenylene, (C_6_H_2_(OH)_2_)_2._

^c^Product_2: Benzene-ether, C_6_H_3_(OH)_2_OC_6_H_3_(OH)_2._

**Table 2:** Element components in the 2D carbon crystal film of 4–6 carbophene.

(In atomic percentige %)* Element	Carbon	Oxygen	Silicon	Copper	Hydrogen
Location 1	95.25	3.128	1.310	0.312	0.000
Location 2	96.25	2.528	0.110	1.114	0.000
Location 3	94.74	3.253	1.248	0.764	0.000
Location 4	96.19	3.082	0.344	0.386	0.000

^*^Semi-quantitative analysis using EDS (energy dispersive spectrometry) method provided by SEM instrument (IXRF, 550i).

**Table 3:** The wavenumbers and intensities of the six gauss peaks in the Raman spectrum of the 4–6 carbonphene crystal.

No.	High a	Center b	Wide σ
Gauss_1	9960	1018	52
Gauss_2	10900	1210	65
Gauss_3	11300	1330	70
Gauss_4	9900	1580	60
Gauss_5	5300	1850	120
Gauss_6	3100	2200	350
