# Supplementary material for: A new type of two-dimensional carbon crystal prepared from 1,3,5-trihydroxybenzene
Source: Sci Rep. 2017 Jan 17;7:40796. doi: 10.1038/srep40796 (PMC5240129; doi:10.1038/srep40796)
Supplement: Supplementary Information [file srep40796-s1.doc]

**Supplementary Information**

**A new type of two-dimensional carbon crystal prepared from** **1,3,5-trihydroxybenzene**

Qi-Shi Du, Pei-Duo Tang, Hua-Lin Huang, Fang-Li Du, Kai Huang, Neng-Zhong Xie, Si-Yu Long, Yan-Ming Li, Jie-Shan Qiu, Ri-Bo Huang

^1^ State key Laboratory of Bioenergy Enzyme Technology, National Engineering Research Center for Non-food Biorefinery, Guangxi Academy of Sciences, Nanning, Guangxi 530007, China

^2^ Institute of Carbon Materials, Dalian University of Technology, No.2 Linggong Road, Ganjingzi District, Dalian, Liaoning, China, 116024

^3^ Gordon Life Science Institute, 53 South Cottage Road, Belmont, MA 02478, USA

**SI 1. Synthesis Experiments**

The synthesis reaction of 4-6 carbophene from 1,3,5-trihydroxybenzene is performed in a quartz tube furnace, as shown in Fig S1.

Figure S1. The quartz tube furnace for synthesis of 2D carbon crystal film of 4-6 carbophene from 1,3,5-trihydroxybenzene.

The copper foil was first polished mechanically and chemically, then was cleaned with acetone, isopropyl alcohol, and deionized water, subsequently dried with argon gas. The quartz glass sheet was washed with acetone, isopropyl alcohol, and deionized water. The γ-aluminum oxide (γ-Al_2_O_3_) was heated at 350 °C for 2 hours. In each of the two ceramic boats 2g of 1,3,5-trihydroxybenzene and 8g of γ-Al_2_O_3_ are loaded, and mixed uniformly. Then one ceramic boat was covered by a copper foil, and the other ceramic boat was covered by a quartz glass sheet. The two ceramic boats were put in the center of quartz tube furnace.

The quartz tube furnace was pumped to vacuum (-0.1 mPa), keeping the low pressure for 10 minutes. Then the quarts tube was filled with argon to ordinary pressure. Above operation was repeated one more time. In the heating procedure 20 sccm argon flow was used as the atmosphere gas. Turning on the heating power, the temperature was increased from room temperature to 350°C in 30 minutes, keeping the temperature for 45 minutes. Then the furnace was cool down to room temperature.


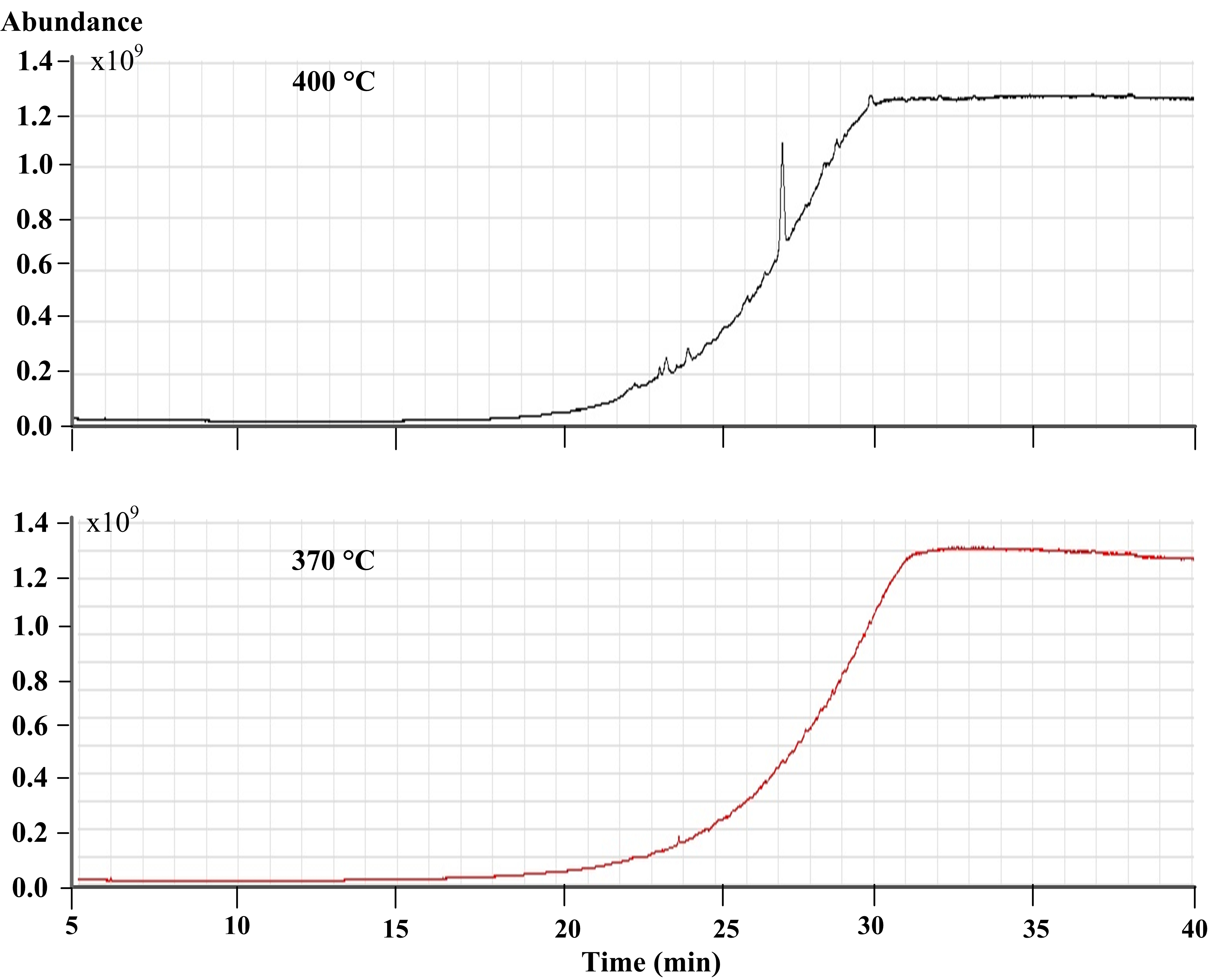


Figure S2. The GC-MS analysis results of the residual gases from synthesis reactions for 4-6 carbophene at 400 ºC and 370 ºC.

The experiments were performed at temperature 350°C, 370°C, 400°C, and 450°C, and the residual gases were absorbed by alcohol, then were analyzed using GC-MS. The analysis results of GC-MS show that when the temperature was lower than 370°C, there basically were no organic compounds in the residual gas; when the temperature was 400°C or high, some organic compounds were detected, indicating the thermal cracking of the 1,3,5-trihydroxybenzene. The results of GC-MS are shown in Fig S2.

After the quartz glass sheet and copper foil were taken out from the quartz tube, the quartz glass and copper foil were washed by 10% sulfate acid solution, acetone, isopropyl alcohol, and deionized water. The 4-6 carbophene films coated on the quartz glass sheet were separated by solvent (N,N-dimethyl formamide). The copper foil was dissolved in FeCl_3_+HCl solution completely. In this way a whole film of the 4-6 carbophene was separated, as shown in Fig S3.

Figure S3. The 4-6 carbophene films separated from the copper foils. The 4-6 carbophene films are coated on the copper foils. After the copper foil is dissolved in FeCl_3_+HCl solution, the 4-6 carbophene film is separated completely.

**SI 2. QM calculations**

There are two possible reaction paths. In the first reaction path two water molecules are dehydrated from two 1,3,5-trihydroxybenzene molecules, and a biphenylene molecule is produced. In the second possible reaction, one water molecule is dehydrated from two 1,3,5-trihydroxybenzene molecules, and a benzene-ether molecule is produced. The thermodynamic properties of the two possible reaction paths are computed using DFT method B3LYP/6-311+G(d,p) at 350 °C (623 K), and the results of reactants and products are listed in Table S1. The QM calculation reports of all reactant and product molecules are stored in file “Free Energy of Molecules.zip”.

Table S1. The calculated inner energies, enthalpy, and free energies of two possible reaction paths from reactant 1,3,5-trihydroxybenzene (B3LYP/6-311+G(d,p), 350 °C).

| Molecules | ΔU° (a.u.)^a^ | ΔH°(a.u.) | ΔG°(a.u.) |
| --- | --- | --- | --- |
| C_6_H_3_(OH)_3_ | -457.751473 | -457.749500 | -457.866678 |
| Product_1^b^ | -762.648472 | -762.646499 | -762.811336 |
| Product_2^c^ | -839.102622 | -839.100649 | -839.283390 |
| Al_2_O_3_ | -710.521939 | -710.519966 | -710.609449 |
| Al(OH)_3_ | -470.026849 | -470.024876 | -470.119574 |
| H_2_O | -76.389182 | -76.387209 | -76.438032 |
|  | | | |
|  | ΔU° (kCal/mol) | ΔH°(kCal/mol) | ΔG°(kCal/mol) |
| Reaction_1 | -104.61 | -107.49 | -103.19 |
| Step_1_1 | 47.76 | 46.52 | 28.84 |
| Step_1_2 | -152.36 | -154.02 | -132.03 |
| Reaction_2 | -69.19 | -65.60 | -58.53 |
| Step_2_1 | 6.99 | 11.40 | 7.49 |
| Step_2_2 | -76.18 | -77.01 | -66.02 |

^a^ 1 a.u. = 627.509469 kCal/mol = 2626.754637 kJ/mol

^b^ Product_1: Biphenylene, (C_6_H_2_(OH)_2_)_2_

^c^ Product_2: Benzene-ether, C_6_H_3_(OH)_2_OC_6_H_3_(OH)_2_

**2.1 Reaction_1**

The first reaction path includes two reaction steps. In the step_1 two water molecules are dehydrated from two 1,3,5-trihydroxybenzene molecules, and a biphenylene molecule is produced. Then in the step_2 the water molecules are absorbed by dehydrant aluminum oxide.

**Step_1**: 2C_6_H_3_(OH)_3_ → (C_6_H_2_(OH)_2_)_2_ + 2H_2_O


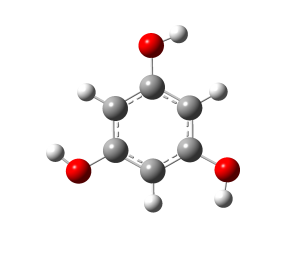

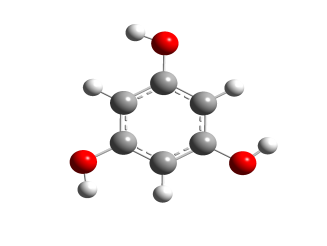


**+**

**→**


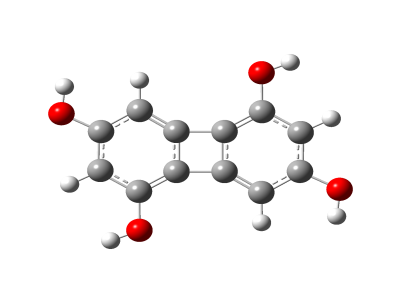


**+**

**2H_2_O**

**Step_2**: Al_2_O_3_+3H_2_O → 2Al(OH)_3_

The detailed QM calculations of the thermodynamic properties of the first reaction path are as follows.

**Step_1_1**

ΔU°R1_1 = [-762.648472-76.389182×2] – [-457.751473×2] = -915.426839 + 915.502946

= 0.076107 a.u. = 47.76 kCal/mol

= 199.9144152 kJ/mol

ΔH°R1_1 = [-762.646499-76.389182 ×2] – [-457.749500×2] = -915.424863 + 915.499000

= 0.074137 a.u.= 46.52 kCal/mol

= 194.737085 kJ/mol

ΔG°R1_1 = [-762.811336-76.438032 ×2] – [-457.866678×2] = -915.6874 + 915.733356

= 0.045956 a.u. = 28.84 kCal/mol

= 120.7151361 kJ/mol

**Step_1_2**

ΔU°R1_2 = [-470.026849×2] – [-710.521939-76.389182×3] = -940.053698 + 939.689485

= -0.364213 a.u. = 228.55 kCal/mol (ΔU°R1_2(H_2_O) = -76.18 kCal/mol)

= -956.698 kJ/mol (One H2O: ΔU°R1_2(H_2_O) = -318.90 kJ/mol)

ΔH°R1_2 = [-470.024876×2] – [-710.519966-76.387209×3] = -940.049752 + 939.681593

= 0.368159 a.u. = 231.023 kCal/mol (ΔH°R1_2(H_2_O) = -77.01 kCal/mol)

= -967.063 kJ/mol (One H2O: ΔH°R1_2(H_2_O) = -322.35 kJ/mol)

ΔG°R1_2 = [-470.119574 ×2] – [-710.609449-76.438032×3] = -940.239148 + 939.923545

= -0.315603 a.u. = 198.04 kCal/mol (ΔG°R1_2(H_2_O) = -66.015 kCal/mol)

= -829.012 kJ/mol (One H2O: ΔG°R1_2(H_2_O) = -276.34 kJ/mol)

The inner energies, enthalpy, and free energies of Reaction_1 are calculated as follows.

ΔU°_R1_ = ΔU°R1_1 +ΔU°R1_2 = 199.91 – 318.90×2 = -437.89 kJ/mol = -104.61 kCal/mol

ΔH°_R1_ = ΔH°R1_1 +ΔH°R1_2 = 194.74 – 322.35×2 = -449.96 kJ/mol = -107.49 kCal/mol

ΔG°_R1_ = ΔG°R1_1 +ΔG°R1_2 = 120.71 – 276.34×2 = -431.97 kJ/mol = -103.19 kCal/mol

The Gibbs free energy of Reaction_1 is -103.19 kCal/mol, therefore it is a spontaneous reaction, and its product is the 4-6 carbophene, as illustrated in Fig S4. In the 2D crystal of 4-6 carbophene the only component element is carbon, and there are no oxygen atoms.


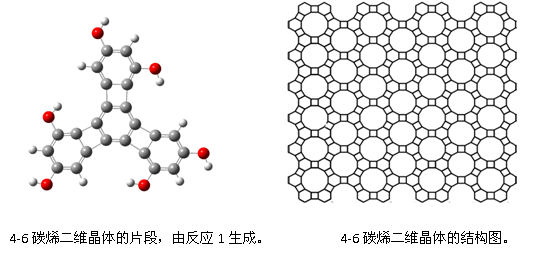


Figure S4. The fragment of 4-6 carbophene (left) and the structure of 4-6 carbophene (right), produced from the first reaction path.

**2**.2 **Reaction_2**

The second reaction path includes two reaction steps. In the step_1 one water molecule is dehydrated from two 1,3,5-trihydroxybenzene molecules, and benzene-ether molecule is produced. Then in the step_2 the water molecule is absorbed by dehydrant aluminum oxide.

**Step_1**: 2C_6_H_3_(OH)_3_ →C_6_H_3_(OH)_2_O C_6_H_3_(OH)_2_ + H_2_O


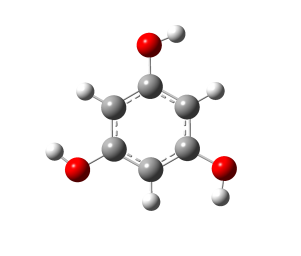

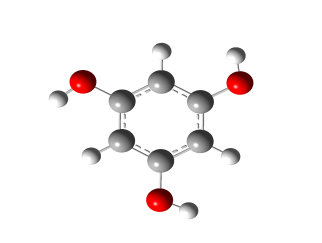


**+**

**→**

**+**

**H_2_O**


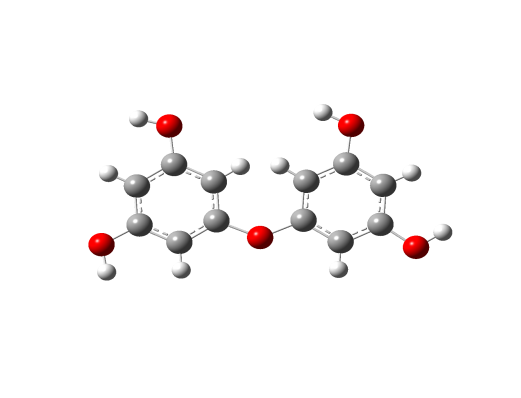


**Step_2**: Al_2_O_3_+3H_2_O → 2Al(OH)_3_

The detailed QM calculations of the thermodynamic properties of the second reaction path are as follows.

**Step_2_1**

ΔU°R2_1 = [-839.102622-76.389182] – [-457.751473×2] = -915.491804 + 915.502946

= 0.011142 a.u. = 6.99 kCal/mol

= 29.27 kJ/mol

ΔH°R2_1 = [-839.100649-76.389182] – [-457.749500×2] = -915.480831 + 915.499000

= 0.018169 a.u. = 11.40 kCal/mol

= 47.73 kJ/mol

ΔG°R2_1 = [-839.283390-76.438032] – [-457.866678×2] = -915.721422 + 915.733356

= 0.011934 a.u. = 7.49 kCal/mol

= 31.35 kJ/mol

**Step_2_2**

ΔU°R2_2 = [-470.026849×2] – [-710.521939-76.389182×3] = -940.053698 + 939.689485

= -0.364213 a.u. = 228.55 kCal/mol (ΔU°R1_2(H_2_O) = -76.18 kCal/mol)

= -956.698 kJ/mol (One H2O: ΔU°R1_2(H_2_O) = -318.90 kJ/mol)

ΔH°R2_2 = [-470.024876×2] – [-710.519966-76.387209×3] = -940.049752 + 939.681593

= 0.368159 a.u. = 231.023 kCal/mol (ΔH°R1_2(H_2_O) = -77.01 kCal/mol)

= -967.063 kJ/mol (One H2O: ΔH°R1_2(H_2_O) = -322.35 kJ/mol)

ΔG°R2_2 = [-470.119574 ×2] – [-710.609449-76.438032×3] = -940.239148 + 939.923545

= -0.315603 a.u. = 198.04 kCal/mol (ΔG°R1_2(H_2_O) = -66.015 kCal/mol)

= -829.012 kJ/mol (One H2O: ΔG°R1_2(H_2_O) = -276.34 kJ/mol)

The inner energies, enthalpy, and free energies of Reaction_2 are calculated as follows.

ΔU°_R2_ = ΔU°R2_1 +ΔU°R2_2 = 29.27 - 318.90 = -289.63 kJ/mol = -69.19 kCal/mol

ΔH°_R2_ = ΔH°R2_1 +ΔH°R2_2 = 47.73 - 322.35 = -274.62 kJ/mol = -65.60 kCal/mol

ΔG°_R2_= ΔG°R2_1 +ΔG°R2_2 = 31.35 - 276.34 = -244.99 kJ/mol = -58.53 kCal/mol

The final product of the second reaction path is the benzene-ether 2D crystal, as illustrated in Fig S5. The benzene-ether 2D crystal consists of two component elements, carbon and oxygen.


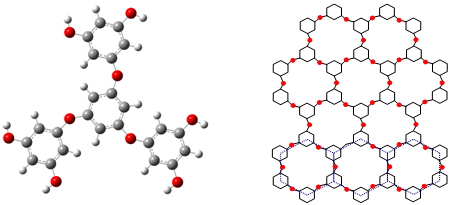


Figure S5. The fragment of benzene-ether 2D srystal (left) and the structure of benzene-ether 2D srystal (right), produced from the second reaction path.

**SI 3. QM calculation reports**

The inner energies, enthalpy, and free energies of all reactant molecules and product molecules are calculated using DFT B3LYP/6-31+G(d,p) method, and the calculation reports are stored in a separated file “Free Energies of Molecules.zip”.
